# Correlation between the Quantity and Type of Dietary Fiber with the Activity in Mexican Patients with Ulcerative Colitis (UC)

**DOI:** 10.3390/nu16183198

**Published:** 2024-09-21

**Authors:** Sophia Eugenia Martínez-Vázquez, José Miguel Corral-Ceballos, Jesús K. Yamamoto-Furusho

**Affiliations:** 1 Department of Gastroenterology, Instituto Nacional de Ciencias Médicas y Nutrición “Salvador Zubirán”, Ciudad de México 14080, Mexico; sophia.martinezv@incmnsz.mx (S.E.M.-V.); josemiguelcoce122@gmail.com (J.M.C.-C.); 2 Inflammatory Bowel Disease Clinic, Instituto Nacional de Ciencias Médicas y Nutrición “Salvador Zubirán”, Vasco de Quiroga 15, Col. Belisario Domínguez Sección XVI, Alcaldía Tlalpan, Ciudad de México 14080, Mexico

**Keywords:** ulcerative colitis, remission, dietary fiber, insoluble fiber

## Abstract

**Background/Objective**: Ingestion of dietary fiber can influence in the remission of patients with ulcerative colitis (UC). There are no current recommendations for fiber intake in UC; therefore, we evaluate the association between dietary fiber and the activity of the disease. **Methods**: Ours is a cross-sectional study in patients with a confirmed diagnosis of UC to whom a 24 h recall was applied; this allowed for the estimation and classification of type of dietary fiber. The patients were divided into two groups: (1) remission and (2) active UC. We analyzed the quantity and type of fiber with the grades of disease activity through Spearman correlation and logistic regression. **Results**: A total of 152 patients were included; it was found that those with clinically active UC consumed less total fiber (*p* = 0.016) and insoluble fiber (*p* = 0.018). Meanwhile, in endoscopic grade, the difference was for insoluble fiber (*p* = 0.038). Insoluble fiber had an inversely significant correlation with fecal calprotectin levels (r = −0.204; *p* = 0.018). Logistic regression showed that less than 11 g of insoluble fiber was a risk factor for clinical activity (OR = 2.37; 95% CI 1.107–5.019; *p* = 0.026). **Conclusions**: Consumption below the current recommendation of total and insoluble dietary fiber is associated with clinical activity of UC.

## 1. Introduction

Chronic idiopathic ulcerative colitis (UC) is the most common subtype of inflammatory bowel disease (IBD), characterized by recurrent inflammation of the mucosa and submucosa of the colon and rectum [1,2]. Currently, IBD has become a global public health problem, to the extent that in 2019, approximately 4.9 million cases were reported [3]. In Mexico, a fourfold increase in the incidence and prevalence of IBD has been observed over a 15-year period, with UC predominating over Crohn’s disease [4]. In other study in 2015, the prevalence of UC was predicted to be around 27.7/100,000 in women and 26.9/100,000 in men, with around 48% of people with UC having moderate-to-severe disease activity [5].

One of the factors contributing the development of the disease is diet and its components; however, as in the case of fiber, there is limited evidence. Dietary fiber is defined as carbohydrate polymers with three or more monomeric units that are not hydrolyzed by endogenous enzymes in the human small intestine [6,7]. It can be classified by many characteristics: solubility (soluble and insoluble), fermentability (highly fermentable and non-fermentable), viscosity (viscous and non-viscous), and structure (short-chain and long-chain carbohydrates) [8].

The fermentation of fiber, especially fermentable soluble fiber, can qualitatively and quantitatively influence the intestinal microbiota, obtaining energy through the production of short-chain fatty acids (SCFAs), promoting the growth of beneficial bacteria (*Lactobacillus* and *Bifidobacterium*) and reducing others (*E. coli*, *Clostridium*) for intestinal homeostasis [6,7]. References to dietary fiber intake are based on energy intake recommendations. The American Dietetic Association has recommended since 2014 that fiber intake for adults must be around 25–30 g per day or 10–14 g per 1000 kcal consumed [8]. Several studies have investigated the relationship between dietary and/or supplemented fiber intake and clinical disease activity, suggesting that fiber is associated with reduced symptoms, clinical activity scores, and C-reactive protein, as well as increased fecal butyrate levels and the relative abundance of *Faecalibacterium prausnitzii* [9,10,11,12,13,14,15]. In a previous report, we concluded that the recommended intake of total fiber had a protective effect on disease activity in Mexican patients [16], but we were unable to determine if the type of fiber influenced this effect. Therefore, the objective of this work was to determine the relationship between the amount and type of dietary fiber and the activity status in patients with UC.

## 2. Materials and Methods

### 2.1. Type of Study

We conducted a cross-sectional study on a cohort of people with a confirmed diagnosis of chronic idiopathic ulcerative colitis to whom a dietary questionnaire was applied.

### 2.2. Population

This study included patients over 18 years old with a diagnosis confirmed by histopathology of UC belonging to the Inflammatory Bowel Disease Clinic at Instituto Nacional de Ciencias Médicas y Nutrición “Salvador Zubirán” (INCMNSZ) between October 2019 and June 2023. All participants signed informed consent and were given a dietary questionnaire. Those who had partial or total colectomy or were consuming any type of supplement or phytotherapy and those who did not agree to participate in the study were excluded.

### 2.3. Procedure

A 24 h food recall was applied and was filled out by the interviewer to avoid interpretation biases and information omission; the application required approximately 30–40 min. Fiber consumption was manually estimated and typified through food and food product composition tables (condensed version 2015) [17].

### 2.4. Evaluation of UC Activity

Clinical activity was evaluated using the Truelove–Witts index, considering the remission or activity of the disease. The Mayo endoscopic score was used to assess endoscopic activity, and fecal calprotectin with a cutoff point greater than 250 µg/g was used for biochemical activity. Inflammation parameters considered in this study were bloody stools (clinically), hemoglobin and fecal calprotectin (biochemically), and the presence of erythema, lack of vascular pattern, friability, erosions, and ulcerations (endoscopically).

### 2.5. Variables Studied

Current age, age at diagnosis, sex, years of evolution, dietary fiber consumption, clinical, biochemical, endoscopic activity of UC, and medical treatment were evaluated.

### 2.6. Sample Size

The cohort of patients with UC was approximately 528 patients at the National Institute of Medical Sciences and Nutrition “Salvador Zubirán”. Using the formula to estimate the proportion, with a 95% confidence interval and 1- effect size, considering that the effect of fiber as a supplement plus mesalazine in prolonging remission reported was 63% by the Fernández-Bañares group et al. The calculated number of individuals was 154 for this study using the Epidat 4.2 online software.

### 2.7. Ethical Procedures

Informed consent was obtained from all subjects involved in the study. Both the consent and the protocol were approved by Ethics Committee of Instituto Nacional de Ciencias Médicas y Nutrición “Salvador Zubirán”, with number registration GAS-3247. Participants were assigned a file number to keep the data and protect the confidentiality of the information. This study was conducted according to the guidelines of the Declaration of Helsinki, maintaining anonymity, privacy, and volition from all the participants.

### 2.8. Statistical Analysis

The population was described with mean, median, percentiles, and frequencies. The population was divided according to the disease period into activity or remission, and based on this, the differences were evaluated with the Mann–Whitney U test and Kendall’s Tau-b. For those with activity, they were classified according to types of activity, and through Spearman correlation, their direction could be determined. Binary logistic regression allowed us to observe the association of both the amount and type of fiber with the type of disease activity.

## 3. Results

**Figure 1:**
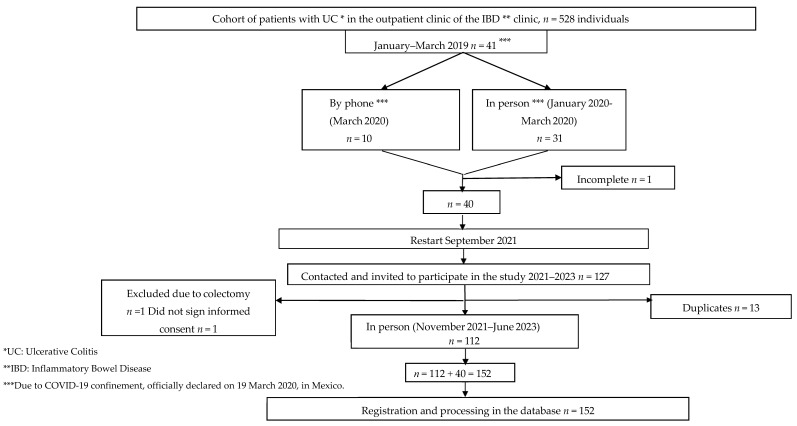
Selection diagram of people with chronic idiopathic ulcerative colitis.

The flowchart of patients included in the study is shown in Figure 1. Of the 152 UC patients included, 51% were women. Most, i.e., 59.2% (*n* = 90), had active UC, and 81% had mild activity. The main sources of fiber were cereals, vegetables, fruits, and legumes, especially insoluble fiber from fruits (41.2 ± 23–5%), vegetables (34.9 ± 24.6%), legumes (30.3 ± 22%), and cereals (29.7 ± 24–6%). We observed that 93% of the studied population consumed cereals, 86% vegetables, 80% fruits, and 25% legumes.

Table 1 shows that the group with active disease had a higher number of years of disease duration (*p* = 0.03), a higher number of relapses in the last year (*p* = 0.000), and higher levels of fecal calprotectin (*p* = 0.000). From the collected dietary data, there were no significant differences in energy, protein, fat, carbohydrates, and free or added sugars. The remaining analysis of the diets including micronutrients can be found in Supplementary Material (Table S1). Regarding fiber, although there were no differences between the groups by disease activity, we decided to evaluate by grade of activity, and Table 2 shows the grams of each type consumed. Each grade of activity also had significant differences, such as bloody stools (*p* = 0.000) and any degree of anemia (*p* = 0.008). According to the Truelove–Witts index, 73 patients had mild activity (*p* = 0.000). At the time of the study, the medications most consumed by patients, in decreasing order, were 5-aminosalicylic acid in 91.4%, thiopurines in 40.1%, steroids in 30.9%, and biologics in 5.9%. The use of monotherapy was present in 47.4% and combination therapy of two drugs in 31.6%, three drugs in 16.4% or four drugs in 1.3%. Among pharmacological treatments, 5-aminosalicylic acid as a monotherapy was the most used in 45.4%, 5-ASA combined with thiopurines in 18.4%, and 5-ASA and steroids in 16.4%.

The grade of UC activity was divided in those who consumed total and insoluble fiber, and significant differences were found between clinical and endoscopic activity, as shown in Table 2. It is noteworthy that those patients in remission consumed around 4 g more total fiber than the recommendation adjusted for energy intake (14 g/1000 kcals), while those with disease activity were slightly below this (−0.2 to 2 g).

**Figure 2:**
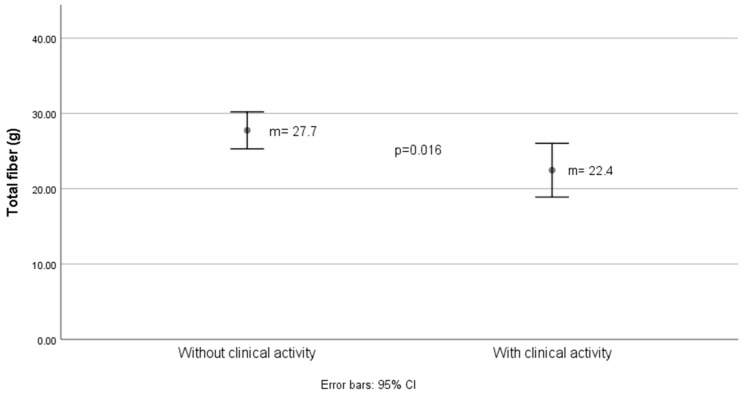
Grams of total fiber estimated in the diet according to the activity status of ulcerative colitis.

**Figure 3:**
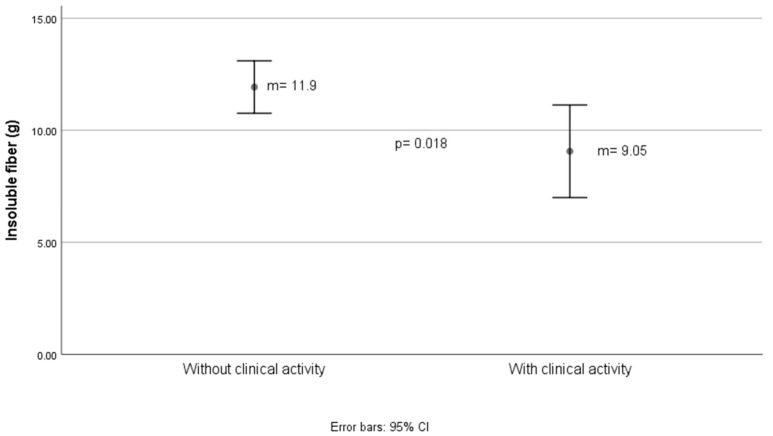
Grams of insoluble fiber estimated in the diet according to the activity status of ulcerative colitis.

Figure 2 and Figure 3 showed differences in total and insoluble fiber intake, respectively, estimated with Mexican food composition tables, for clinical activity determined by 2 characteristics: relapses in the last year and the Truelove–Witts index.

The difference in insoluble dietary fiber intake between groups with and without clinical activity was 3 g, as shown in Figure 3.

**Figure 4:**
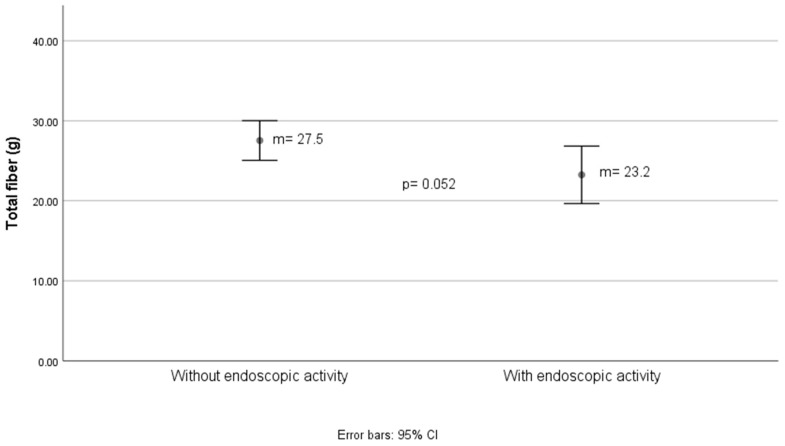
Grams of total fiber estimated in the diet according to the endoscopic activity status of ulcerative colitis.

**Figure 5:**
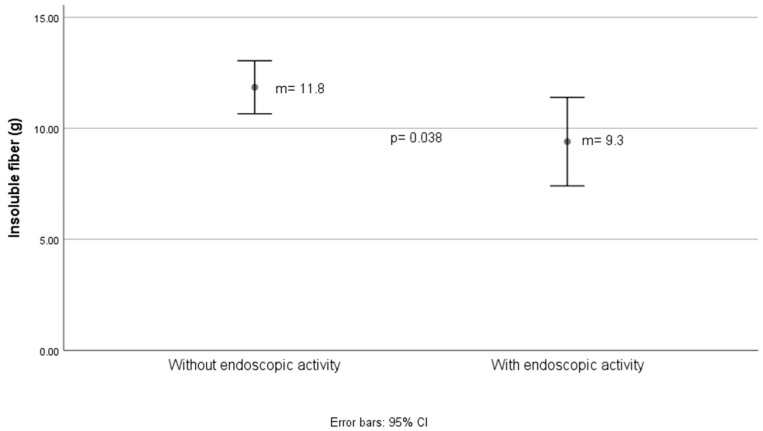
Grams of insoluble fiber estimated in the diet according to the endoscopic activity status of ulcerative colitis.

Figure 4 and Figure 5 show differences in total and insoluble fiber intake, respectively, estimated with Mexican food composition tables, for endoscopic activity determined by the Mayo score (which for the purposes of this study was divided into present and absent). For total fiber, the difference was approximately 4 g between groups. For insoluble dietary fiber, the difference was 2.5 g, as shown in Figure 5.

### 3.1. Correlation Analysis

The analysis confirmed weak correlations, which were significant between total and insoluble fiber and clinical and endoscopic activity, as shown in Table 3.

**Figure 6:**
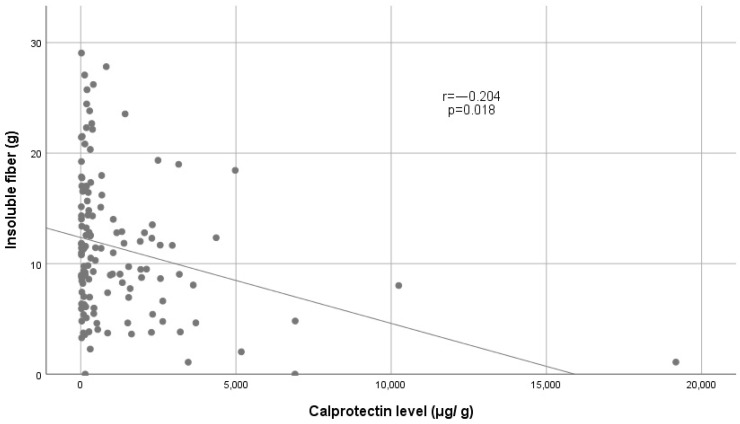
Correlation of fecal calprotectin level with grams of insoluble fiber.

In particular, upon performing this type of analysis, we found that insoluble fiber had a significantly inversely proportional correlation (r = −0.204, *p* = 0.018) with fecal calprotectin levels; specifically, the fiber ranged between 4 and 18 g, as shown in Figure 6.

### 3.2. Regression Analysis

Based on the median total and insoluble fiber intake by disease activity groups, cutoff points were proposed to evaluate the association of fiber types with the degree of UC activity. For total fiber, the cutoff point was established at 24 g per day, while for insoluble fiber, the cutoff point was 11 g per day. Soluble fiber was not evaluated because it did not show significant differences in the univariate analysis. The results showed that consuming less than 11 g per day of insoluble fiber and 24 g per day of total fiber were risk factors for clinical activity (β = 2.37, *p* = 0.026 and β = 2.039, *p* = 0.058, respectively), as shown in Table 4.

## 4. Discussion

In this study, we found an inverse relationship between the amount of habitual dietary fiber and clinical, biochemical, and endoscopic activity in patients with UC. The vast majority of patients had mild activity with a higher number of relapses, anemia, and elevated fecal calprotectin levels compared to those patients with UC in remission. Total fiber intake was significantly lower in patients with clinical and endoscopic activity, while insoluble fiber was significantly higher among those without clinical, biochemical, and endoscopic activity.

The results of this study showed differences between total fiber intake (5.3 g) and insoluble fiber (2.85 g) between groups with and without clinical activity. Similarly, differences were observed regarding total fiber intake (4.3 g) and insoluble fiber (2.5 g) with the endoscopic activity of UC. It is noteworthy that the amount of fiber reported among those with activity was slightly below the current recommendations for the general population, but not as low as the average intake reported in other groups of patients with UC, which is around 12 to 17 g per day [18,19]. Therefore, we believe that UC is sensitive to small dietary modifications, as observed with pharmacological treatment, suggesting that adding 5 g of fiber to the diet may maintain disease remission. We know that this relationship exists between fiber from the diet and not from supplements because supplements were an exclusion criterion, and in our center, fiber supplementation is not a common maneuver for treating disease, so patients had not received a prescription or exposure to it. We suggest that fibers from food cannot be replaced with fiber supplements before another study is conducted because of the possible anti-inflammatory or antioxidant activity of other food components.

The correlation between the clinical and endoscopic activities of UC with both total and insoluble fiber was low but statistically significant. Insoluble fiber was inversely correlated with fecal calprotectin levels, suggesting that consuming 10 to 12 g of this type of fiber may have some benefit in UC activity, supporting that half of the dietary fiber intake should be of this type. Previous studies have proposed that the principal role of this type of fiber is the formation of soft and bulky stools that are easy to evacuate by increasing their water content, improving regulation in bowel movements. This process occurs due to the irritating effect on the mucosa of the large intestine, which stimulates water and mucus secretion [20]. The classification by solubility, however, does not provide a greater advantage in understanding the functional effect that could explain its association with cardiovascular diseases, diabetes, and some types of cancer [21]. According to the literature reporting types of fiber by their solubility, fermentability, and viscosity, it is known that insoluble dietary fiber is non-viscous but may have a laxative effect or cause constipation, a situation that depends on the particular food source (such as cellulose from fruits and vegetables or wheat bran) [22]. The results of a recent meta-analysis [23] showed no association of dietetic fiber with UC but a significant relation with fruit RR (0.69; 95% CI: 0.55, 0.86) and with vegetable RR (0.56; 95% CI: 0.48, 0.66), suggesting the intervention of other food components as phytochemicals present in raw food sources. On the other hand, it is not clear whether it is the fermentation of the fiber, the phytochemicals, or their synergy that produces a protective effect. In this study, we did not analyze food sources of insoluble fiber, although we know that the highest percentage of availability of insoluble fiber in these patients was from cereals, vegetables, and fruits, and only a quarter of the studied population obtained it from legumes.

Until now, this is the first study in Mexico and Latin America which has evaluated the role of fiber in UC patients, suggesting that consuming less than 24 g per day of total fiber and 11 g of insoluble fiber in patients with UC are risk factors for the clinical, biochemical, and endoscopic activity of UC.

It is currently known that fiber has a relevant role in the microbiota largely due to the production of short-chain fatty acids (SCFAs) mainly producing butyrate, propionate, and acetate, which are the products of fermentation carried out by the intestinal microbiota through fermentable soluble dietary fiber. Butyrate stands out among SCFAs as it acts as the main energy source for colonocytes, helping to maintain the integrity of the intestinal barrier and thus decreasing intestinal permeability [21]. On the other hand, butyrate along with acetate balances the production and secretion of mucus, which functions at the epithelial level as a protector against pathogens [24]. It is known that fermentable soluble fiber helps in the abundance and diversity of the intestinal microbiota by allowing energy to be obtained through its fermentation, increasing beneficial strains and limiting the growth of harmful ones. This was observed in a study by Fritsch et al. [16], where after intervening with a low-fat and high-fiber diet, the authors observed an increase in β diversity (*p* = 0.05), *Bacteroidetes* (14.6% to 24.02%; *p* = 0.015), and *Prevotella* (0.36% to 0.69%; *p* = 0.0007) compared to the baseline; they also observed an increase in *F. prausnitzii* (5.37% to 7.2%; *p* = 0.04) compared to an improved standard American diet. Keshteli et al. [25] in a randomized, open-label placebo-controlled trial using two dietary interventions (anti-inflammatory diet vs. Canada’s food guide) found an increase from the beginning to the end of the trial in the abundance of certain bacteria like *Bifidobacteriaceae*, *Lachnospiraceae*, and *Ruminococcaceae*, proposing that they could contribute to the protective mechanism of the anti-inflammatory diet associated with the relapse phase of UC. They also found that increased consumption of anti-inflammatory foods correlated with a significant increase in *Collinsella* and *Blauti*, and as has been described, *Blautia* is among the microorganisms associated with decreased colonic inflammation.

While fiber solubility partly determines the physiological effects it exerts on the colon, other factors that influence fiber functionality, such as fermentability and the viscous capacity for gel formation, are currently recognized [21]. The influence of various bioactive components; food processing and preparation; the interaction of nutrients with each other and with the environment; and their true availability for the individual and their microbiota still needs to be explored. Methods for evaluating diet overlook this complexity, as diet and food should not be viewed from a reductionist perspective as the intake of a single nutrient. We consider this a limitation in the analysis of our data. The possibility of analyzing food matrices and fiber sources could allow for a better explanation of their impact and understanding of their association with disease periods. Another limitation we found is the food record, which could not be representative of the usual diet. We outline a third limitation, which is the associations with clinical, endoscopic activities, and calprotectin, but not with histological activity, which does not mean that it did not have any impact; that said, it was not possible to confirm in this study. Finally, we recognize that the exposure to pharmacologic therapy was not completely the same in the analyzed groups, which could also have influenced the intestinal environment.

In conducting this study, we had successes in estimating and typifying dietary fiber in people with this disease, factors that were as yet unknown in our environment. This allowed us to better guide people with this disease, and we hope it will be useful in clinical practice as some healthcare centers prohibit fiber-containing diets in managing this disease. On the other hand, determining which type of fiber should predominate highlights that both insoluble and soluble fiber should be prescribed and that classification by solubility should not be the only criterion in doing so; viscosity, fermentability, and the food source and matrix should also be considered.

## 5. Conclusions

Consumption of total and insoluble dietary fiber below the current recommendations correlated with clinical, biochemical, and endoscopic activity in Mexican patients with UC. Higher consumption of dietary fiber might help to maintain disease remission.

## Figures and Tables

**Table 1:** Demographic characteristics of the studied population according to disease activity.

Characteristics	Population Med (P25–75)	Active (*n* = 90) Med (P25–75)	Remission (*n* = 62) Med (P25–75)	*p* Value
Age	41 (31–57)	41 (38–45)	43 (35–55)	0.185
Gender (Female)	78 (51.3%)	52 (66.6%)	26 (33.3%)	0.055
Years of evolution	10 (5–17)	8.5 (6–13)	11.5 (9–13)	0.037
Total relapses in the last year	16 (10.5%)	9 (10%)	7 (11.29%)	0.000
Calprotectin	294.4 (91.8–1451.05)	176.9 (44–144.6)	1025.3 (519–1543.6)	0.000
Kcal	1723.42 (1305.23–2288.46)	1718 (1545–1826.8)	1747.5 (1507–1976.7)	0.728
Proteins (g)	75.6 (58.13–93.39)	75.64 (64.7–82.8)	75.7 (67.39–84.3)	0.747
Fats (g)	40.57 (27.05–55.37)	40.7 (34.9–45.2)	39.92 (35.7–47)	0.455
HC (g)	217.58 (162.24–305.52)	223.9 (197.2–255.9)	212.3 (174.8–241.8)	0.606
Free sugars	49.83 (34–79.06)	50.86 (43.1–64.4)	48.44 (44.4–60.8)	0.184
Total fiber	23.86 (16.8–34.1)	23.12 (19.9–25.4)	25.45 (22.3–29.8)	0.079
Insoluble fiber	10.6 (6.4–14.7)	9.05 (8.0–11.6)	11.42 (9.7–14.0)	0.147
Soluble fiber	12.61 (7.0–20.1)	12.42 (10.2–14.4)	13.75 (10.6–16.8)	0.981
Disease Activity				
Clínic	41	41	0	0.000
Endoscopy	43	42	1	0.000
Biochemical	74	73	1	0.000
Number of bloody Stools				
<4/day	131	70	61	0.000
4–6/day	14	13	1	
>6/day	7	7	0	
Anemia				
Mild (>1.5 g/dL)	133	74	59	0.008
Moderate (10.5–11.5 g/dL)	13	11	2	
Severe(<10.5 g/dL)	6	5	1	

Kendall’s Tau-b, HC = carbohydrates.

**Table 2:** Grams of fiber by grade of ulcerative colitis activity.

	Clinic Activity			Endoscopic Activity			Biochemical Activity		
	No	Yes	*p* *	No	Yes	*p* *	No	Yes	*p* *
Total fiber	27.75	22.45	0.016	27.53	23.54	0.052	27.93	24.62	0.111
Insoluble fiber	11.93	9.06	0.018	11.85	9.4	0.038	12.01	10.26	0.094
Soluble fiber	15.82	18.34	0.650	15.68	18.56	0.586	15.92	17.11	0.724

* *t*-Student.

**Table 3:** Correlations between the grade of fiber and the grade of UC activity.

Activity	Dietary Fiber		Insoluble Dietary Fiber		Soluble Dietary Fiber	
	r	*p* Value	r	*p* Value	r	*p* Value
Clínic	−0.192	0.018	−0.215	0.008	−0.093	0.315
Endoscopy	−0.164	0.044	−0.187	0.021	−0.052	0.175
Biochemical	−0.130	0.109	−0.160	0.049	−0.040	0.261

r = Spearman.

**Table 4:** Association between total fiber ^1^ and insoluble fiber ^2^ by type of UC activity.

Activity	Total Fiber β (IC95%)	*p* Value	Insoluble Fiber β (IC95%)	*p* Value
Clinic	2.039 (0.976–4.26)	0.058	2.37 (1.107–5.019)	0.026
Endoscopic	1.91 (0.965–4.10)	0.062	1.97 (0.950–4.097)	0.069
Biochemical	1.61 (0.850–3.60)	0.144	0.615 (0.324–1.17)	0.139

^1^ = <24 g per day, ^2^ = <11 g per day, logistic regression.

## Data Availability

The data presented in this study are available on request from the corresponding author. The data are not publicly available because they contain information that could compromise the privacy of the research participants.

## Supplementary Materials

The following supporting information can be downloaded from https://www.mdpi.com/article/10.3390/nu16183198/s1, Table S1: Other nutrients calculated from 24 h recalls obtained from tables of Mexican food composition [18].
