# Supplementary material for: Correlation between the Quantity and Type of Dietary Fiber with the Activity in Mexican Patients with Ulcerative Colitis (UC)
Source: Nutrients. 2024 Sep 21;16(18):3198. doi: 10.3390/nu16183198 (PMC11435422; doi:10.3390/nu16183198)
Supplement: Supplementary file 1 [file nutrients-16-03198-s001.zip › nutrients-3209804-supplementary.pdf]

Table S1. Nutrients of the studied population according to disease activity.

Nutrients	Median (P25-75)	UC activity		P value
		Active Median (P25-75)	Remission Median (P25-75)	
Calcium (mg)	831.74 (537.39-1113.58)	860.43 (746.97-941.35)	761.78 (642.41-970.39)	0.32
Phosphorus (mg)	1039.05 (790.7-1306.04)	1055.06 (982.95-1186.97)	1004.28 (898.2-1095.99)	0.14
Iron (mg)	22.72 (15.51-32.61)	21.65 (18.42-26.83)	23.52 (20.81-27.5)	0.49
Sodium (mg)	1477.95 (816.63-2196.88)	1477.95 (1153.83-1810.93)	1420.72 (1121.37-1839.67)	0.79
Potassium (mg)	2142.67 (1522.73-3004.14)	2453.93 (2070.82-2804.38)	2099.18 (1888.58-2200.25)	0.15
Magnesium (mg)	279.26(218.6-392.69)	306.03 (276.03-349.86)	267.34 (249.3-298.33)	0.16
Copper (mg)	2.31 (1.65-3.58)	2.69 (2.22-3.08)	2.24 (1.89-2.42)	0.11
Zinc (mg)	13.31 (10.49-19.17)	13.83 (13.2-15.37)	12.5 (11.22-14.28)	0.07
Manganese (mg)	2.1 (1.53-3.02)	2.07 (1.89-2.26)	2.12 (1.9-2.53)	0.99
Selenium (mg)	0.11(0.07-1.01)	0.13 (0.1-0.19)	0.11 (0.09-0.15)	0.86
Lithium (mg)	0	0	0	0.40
Vitamin A IU	2111.46 (1471.21-3424.97)	2239.54 (1989.16-2752.78)	2003.09 (1695.75-2226.6)	0.12
Vitamin A (µg RAE)	322.79 (158.44-631.52)	302.83 (211.65-386.46)	331.59 (256.56-517.77)	0.53
Carotenes (mg)	128.36 (0-665.0.5)	200 (14.55-320.03)	52.8 (0.99-320)	0.35
Beta-carotene (mg)	2.23 (0.39-5.77)	2.73 (1.01-4.83)	2.18 (0.99-3.25)	0.43
Vitamin B1 (mg)	0.96 (0.75-1.39)	1.07 (0.9-1.24)	0.93 (0.85-1.08)	0.2
Vitamin B2 (mg)	1.28 (0.88-2.02)	1.31 (1.12-1.58)	1.26 (1.03-1.47)	0.48
Niacin (mg)	15.36 (11.3-21.59)	15.17 (12.68-18.56)	15.45 (13.79-18.56)	0.69
Ascorbic acid (mg)	135.25 (54.87-585.34)	141.77 (110.98-283.17)	122.3 (89.94-319.98)	0.74
Vitamin B6 (mg)	1.72 (1.21-2.41)	1.75 (1.54-2.1)	1.72 (1.44-2.02)	0.69
Vitamin B12 (µg)	2.51 (1.45-5.46)	2.33 (1.64-3.64)	2.53 (2.19-3.53)	0.59
Folic acid (µg)	64.07 (18.58-119.34)	71.33 (44.08-91.08)	61.67 (38.02-81.6)	0.51
Folate (µg DFE)	329.71 (238.66-502.59)	355.91 (301.37-439.36)	313.15 (286.89-354.22)	0.22
Vitamin D ((µg))	2.32 (0.64-3.31)	2.37 (2.11-2.56)	2.24 (1.64-2.68)	0.86
